# Mitral Annular Kinetics, Left Atrial, and Left Ventricular Diastolic Function Post Mitral Valve Repair in Degenerative Mitral Regurgitation

**DOI:** 10.3389/fcvm.2015.00031

**Published:** 2015-08-17

**Authors:** Chun G. Schiros, Mustafa I. Ahmed, David C. McGiffin, Xiaoxia Zhang, Steven G. Lloyd, Inmaculada Aban, Thomas S. Denney, Louis J. Dell’Italia, Himanshu Gupta

**Affiliations:** ^1^Department of Medicine, Division of Cardiovascular Disease, The University of Alabama at Birmingham, Birmingham, AL, USA; ^2^Alfred Health, Melbourne, VIC, Australia; ^3^Department of Electrical and Computer Engineering, Samuel Ginn College of Engineering, Auburn University, Auburn, AL, USA; ^4^Birmingham Veteran Affairs Medical Center, Birmingham, AL, USA; ^5^Department of Biostatistics, The University of Alabama at Birmingham, Birmingham, AL, USA

**Keywords:** left ventricular diastolic function, left atrial function, mitral valve repair, isolated mitral regurgitation, mitral annular kinetics

## Abstract

**Objective:**

The relationship of mitral annular (MA) kinetics to left ventricular (LV) and left atrial (LA) function before and after mitral valve (MV) repair has not been well studied. Here we sought to provide comprehensive analysis that relates to MA motions, and LA and LV diastolic function post MV repair.

**Methods:**

Three-dimensional analyses of mitral annular motion, LA function, and LV volumetric and diastolic strain rates were performed on 35 degenerative mitral regurgitation (MR) patients at baseline and 1-year post MV repair, and 51 normal controls, utilizing cardiac magnetic resonance imaging with tissue tagging.

**Results:**

All had normal LV ejection fraction (EF) at baseline. LV and LA EFs decreased 1-year post-surgery vs. controls. LV early diastolic myocardial strain rates decreased post-surgery along with decreases in normalized early diastolic filling rate, E/A ratio, and early diastolic MA relaxation rates. Post-surgical LA late active kick remained higher in MR patients vs. control. LV and LA EFs were significantly associated with peak MA centroid to apex shortening. Furthermore, during LV systolic phase, peak LV ejection and LA filling rates were significantly correlated with peak MA centroid to apex shortening rate, respectively. While during LV diastolic phase, both peak early diastolic MA centroid to apex relaxation rate and LA ejection rate were positively significantly associated with LV peak early diastolic filling rate.

**Conclusion:**

MA motion is significantly associated with LA and LV function. Mitral annular motion, left atrial function, and LV diastolic strain rates are still impaired 1 year post MV repair. Long-term effects of these impairments should be prospectively evaluated.

## Introduction

Degenerative mitral regurgitation (MR), usually related to mitral valve (MV) prolapse, is responsible for most cases of isolated MR in the US (1), causing a major public health burden. The natural history of MR is progressive left ventricular (LV) dysfunction and adverse LV remodeling, eventually leading to heart failure. There is no effective medical therapy for isolated MR, and therefore, surgery is recommended in patients with severe MR and symptoms or evidence of progressive LV dysfunction (2, 3). However, we have previously demonstrated that in isolated MR with normal LVEF, despite adherence to the guideline recommendations at the time of operation, there is impaired LV systolic function and persistent spherical remodeling 1-year post surgery (4–6). Furthermore, recent updates to the guidelines allow for surgical repair in selected asymptomatic severe MR patients even if LV end-systolic dimension (LVESD) is <40 mm and LV ejection fraction (EF) >60% (4).

The etiopathogenesis of degenerative MR is due to alteration in any of the major structures constituting the MV apparatus that include MV leaflets, mitral annulus (MA), chordae, and papillary muscles. MA is a saddle-shaped structure that is anatomically and functionally contiguous with both left atrium (LA) and LV.(7–9) It demonstrates complex motion during the cardiac cycle that is mediated predominantly due to contractility of LA and LV (10).

Although MA kinetics plays an important role in coupling LA and LV and is integral to normal LV systolic and diastolic function (10, 11), this relationship has not been well described post MV repair. In this study, we extend our previous work (6) and utilized comprehensive cardiac magnetic resonance imaging (cMRI) with tagging and 3-dimensional (3D) analysis to evaluate mitral annular motion, and LA and LV function.

## Materials and Methods

### Study population

The same study population was used in the current investigation as our previous study (6), consisting of 51 control subjects, 35 patients with moderate to severe MR secondary to degenerative MV disease who were referred for corrective MV repair. Patients underwent surgery based on the conservative clinical judgment of the cardiologist and cardiovascular surgeon at the tertiary referral center. Their LV EF was 61 ± 7% and LV end diastolic dimension 39 ± 6 mm by cMRI. All the patients underwent coronary angiography before surgery to rule out significant coronary artery disease. Patients with evidence of significant aortic valve disease or concomitant mitral stenosis were excluded. In 14 patients, both anterior and posterior MV leaflets were affected. A variety of surgical techniques were used to repair the MV, including leaflet resection, chordal replacement, or a combination of both. All had implantation of flexible Duran annuloplasty bands, the dimension of which ranged from 29 to 35 mm. The adequacy of the repair was assessed by intraoperative transesophageal echocardiography.

The control subjects had no prior history of cardiovascular disease and were not taking any cardiovascular medications. The study protocol was approved by the Institutional Review Boards of University of Alabama at Birmingham. All participants gave written informed content.

### Cardiac MRI (cMRI)

Cine cMR was performed on a 1.5-T MRI scanner (Signa, GE Healthcare, Milwaukee, Wisconsin) optimized for cardiac imaging. Electrocardiographically gated breath-hold steady-state free precision technique was used to obtain standard (2-, 3-, and 4-chamber long axis and serial parallel short-axis) views using the following typical variables: slice thickness of the imaging planes 8 mm with zero inter-slice gap, field of view 40 cm, scan matrix 256 × 128, flip angle 45°, repetition/echo times 3.8/1.6 ms, and number of reconstructed cardiac phases 20. Tagged cMR was done on exact slice prescriptions as cine cMR by applying grid tagging to the short-axis views and stripe tagging to long-axis views using spatial modulation of magnetization encoding gradients method (FGR-SPAMM) as previously described with following general variables: prospective ECG triggering, trigger time 10 ms from *R* wave, slice thickness 8 mm, zero inter-slice gap, field of view 40 cm × 40 cm, scan matrix 256 × 128, flip angle 10°, repetition/echo times 8.0/4.2 ms, views per segment 8–10, tag spacing 7 mm, and number of reconstructed cardiac phases 20.

The methodology of measuring LV volumetric variables was previously described (6). Basal septal, lateral, anterior, and inferior landmarks on the MA as well as LV apex were manually placed on end-diastolic (ED) and ES, and automatically tracked through the cardiac cycle using an in-house dual propagation technique (12). MA septal, lateral, anterior, and inferior displacement vs. time curves were constructed over the cardiac cycle and differentiated with respect to time to obtain the peak early diastolic MA velocities at all regional locations. MA transverse diameter was measured as the distance between the basal anterior and inferior landmarks while the septal-lateral diameter was measured as the distance between the basal septal and lateral landmarks. MA plane centroid was approximated as the mean of the four landmarks. The MA plane was then approximated as a flat plane going through the centroid and optimizing its distance to the four landmarks. The MA plane normal was computed. Plane centroid to apex shortening from the reference ED time frame was computed in each time frame.

LA volumes were computed using biplane area-length method with manual contours on two and four chamber long-axis views (13, 14) for each time frame. LA volumetric measurements were provided as maximum atrial volume V_max_ when MV opens, minimum atrial volume V_min_ when MV closes, and pre-atrial kick volume V_active_, measured at the time of peak LV late filling rate. LA stroke volume was computed as V_max_−V_min._ LA reservoir function was measured as LAEF = (V_max_−V_min_)/V_max_; LA passive emptying function was measured as LAEF_passive_ = (V_max_−V_active_)/V_active_, and LA pump function was measured as LAEF_active_ = (V_active_−V_min_)/V_active_ (15).

Two-dimensional (2D) circumferential and longitudinal strains and strain rates were measured using harmonic phase (HARP) analysis (16). Because the tag lines faded with time due to T_1_ relaxation, 2D circumferential and longitudinal strain was only calculated through 150% of systole. 2D twist *T* at timeframe *t* was measured by tracking a circular mesh of points in a basal and apical slice of that timeframe. The mesh was identified in the first time based on user-defined contours and tracked through the remaining imaged phases using improved HARP tracking (17). Normalized twist was defined as *T*/*L*, where *L* is the longitudinal distance between the basal and distal slices at ED timeframe (18). Peak early diastolic normalized untwist rate was further adjusted to diastolic interval. Torsion shear angle *φ* over the cardiac cycle was computed as

$$\varphi \left(t\right)=T\left(t\right)\times \frac{\rho_{\text{base}}\left(t\right)+\rho_{\text{apex}}\left(t\right)}{2L};$$

where ρ(*t*) is the epicardial radius at timeframe *t* and *L* is the longitudinal distance between the basal and distal slices at ED timeframe. 2D φ*t* curve (torsion shear angle-time curve) was constructed for each subject.

### Statistical analysis

Comparisons of MRI variables among controls and MR patients before and 12 months after surgery were performed using a mixed model via PROC MIXED. The repeated measures of MR patients before and after surgery were accounted for by an assumed compound symmetry correlation structure. Tukey-Kramer test procedure was utilized to adjust the significance level accordingly. Combining control and post-surgical MR groups (*N* = 86), the associations among MA motion, and LV and LA functions were tested during systole and diastole, respectively, using simple and multiple regression models. Simple linear regression was performed between peak MA centroid to apex shortening and LAEF, LVEF, respectively, between peak LV ejection rate and MA centroid to apex shortening rate, and between peak LA filling rate and MA centroid to apex shortening rate. Multiple linear regression was performed using LV peak early diastolic filing rate as dependent variable and both MA peak early diastolic centroid to apex relaxation rate and LA peak early diastolic ejection rate as independent variables. All data were presented as mean ± standard deviation. To account for correlations amongst the variables, a *P* < 0.01 was considered statistically significant for group-wise comparisons. A *P* < 0.05 was considered statistically significant for the regression models. We also conducted a general linear model for functional variables to adjust for age and gender using analysis of covariance. All statistical analyses were performed using SAS version 9.3.

## Results

The clinical characteristics of the MR patients and control subjects are described in Table 1. More detailed characteristics can be found in our previous study (6). The MR patient group was significantly older than the control group with a lower percentage of female. Among the 35 MR patients, nine of them were on beta blocker before surgery and only three were discontinued at 12-month follow-up. Seven patients were on angiotensin-converting-enzyme inhibitors/angiotensin receptor blockers at baseline and five of them were discontinued at the 12-month follow-up. Lastly, five patients were on calcium blocker and four were discontinued 12 month after surgery. Despite reduction in LV volumes of MR 1 year post surgery, it still remained significantly higher than the controls. Before surgery, mitral regurgitant volume was 54 ± 34 ml and regurgitant fraction was 38 ± 16%. One year after surgery, the regurgitant volume was 1.6 ± 14 ml and regurgitant fraction was 2 ± 16%.

**Table 1:** **Clinical characteristics of surgical patients with mitral valve repair**.

	Control (*n* *=* 51)	Mitral regurgitation	
		Pre-operative (*n* *=* 35)	Post-operative (*n* *=* 35)
Age (years)	44 ± 14	53 ± 11*	54 ± 11*
% Female	53	20*	20*
Body surface area (m^2^)	1.9 ± 0.24	2.00 ± 0.24	1.98 ± 0.23
Heart rate (beats/min)	67 ± 12	71 ± 11	69 ± 10
Systolic BP (mm Hg)	118 ± 13	124 ± 15	121 ± 11
Diastolic BP (mm Hg)	75 ± 10	78 ± 8	76 ± 10
LV ED volume index (ml/m^2^)	69 ± 10	112 ± 24*	80 ± 18*^,^^†^
LV ES volume index (ml/m^2^)	25 ± 7	45 ± 13*	38 ± 14*^,^^†^
LV EF (%)	64 ± 7	61 ± 7*	54 ± 8*^,^^†^
LV ED mass index (g/m^2^)	50 ± 10	67 ± 14*	57 ± 13*^,^^†^
Mitral regurgitant volume (ml)		54 ± 34	1.6 ± 14^†^
Mitral regurgitant fraction (%)		38 ± 16	2 ± 16^†^

*Values are *n* or mean ± SD*.

*P < 0.05 vs. control.

*^†^*P* < 0.05 vs. pre-operative MR*.

### LV functional measurements

**Figure 1:**
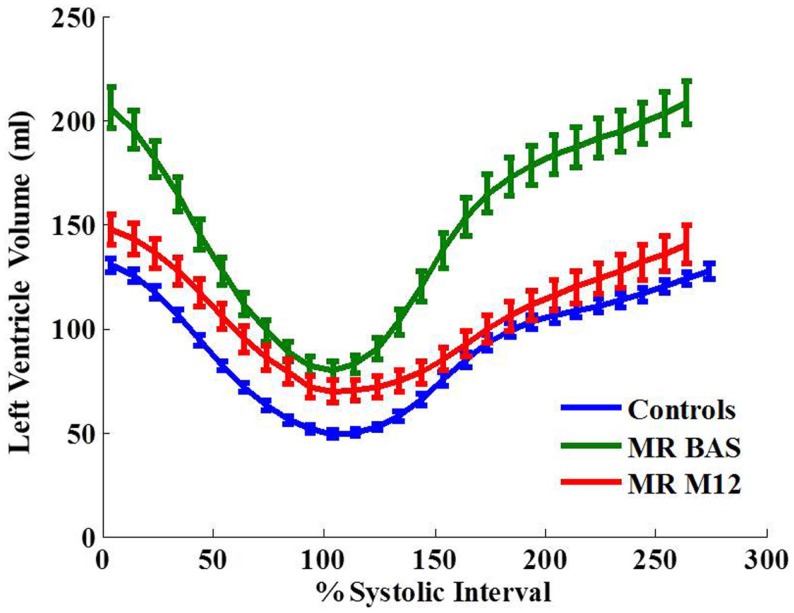
**Left ventricular volume-time curves for the control group and MR group at baseline and 12 months after surgery**. Data are mean ± SE. Ejection rate and early filling rate are decreased in MR 1 year after surgery.

**Figure 2:**
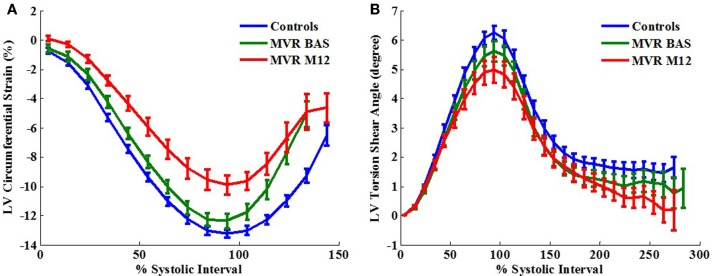
**Two-dimensional circumferential strain vs. time curves (A) and torsion shear angle vs. time curves (B) in control vs. MR before and after surgery**. One year after surgery, MR patients demonstrate a decrease in circumferential strain relaxation rate as represented by a flatter curve during early diastole.

Left ventricular normalized peak ejection rate was significantly decreased after surgery in MR patients (Table 2) which was also demonstrated on the LV volume-time curves depicted in Figure 1. LV peak normalized early diastolic filling rate, E/A ratio, and peak early diastolic circumferential and longitudinal strain rates were significantly decreased in the post-surgical MR group vs. control and pre-surgical MR. After adjusting for age and gender, the difference remained significant for peak normalized early diastolic filling rate. Figure 2A further demonstrates a lower relaxation rate as a flatter circumferential strain vs. time curve during diastole in the MR group post-surgery. On evaluating torsional mechanics of LV, we noted that peak early diastolic normalized untwist rate (adjusted to diastolic interval) remained the same in MR after surgery. LV torsion shear angle over the cardiac cycle in the MR group after surgery vs. controls and pre-surgical MR is as shown in Figure 2B.

**Table 2:** **MRI-derived LV functional measurements**.

	Control (*n* *=* 51)	Mitral regurgitation	
		Pre-operative (*n* *=* 35)	Post-operative (*n* *=* 35)
Peak ejection rate (EDV/s)	3.13 ± 0.45	2.98 ± 0.63	2.77 ± 0.62*^,^^§^
Peak E. Dia. filling rate (EDV/s)	3.13 ± 0.45	3.04 ± 0.78	1.91 ± 0.42*^,^^†^
E/A ratio	2.10 ± 1.04	2.39 ± 1.13	1.39 ± 0.49^†^
Peak E. Dia. Circ. strain rate (%/s)	96 ± 30	99 ± 29	71 ± 30^†,^^‡^
Peak E. Dia. long. strain rate (%/s)	104 ± 32	123 ± 55*	80 ± 33^†^
Peak E. Dia. normalized untwist rate (/cm/%Diastolic interval)	0.09 ± 0.03	0.07 ± 0.02^‡^	0.07 ± 0.04^‡^
Time to peak untwist rate (ms)	385 ± 40	390 ± 39	403 ± 70

*Values are mean ± SD*.

*E. Dia., early diastole; MA, mitral annulus; LA, long axis; Circ., circumferential; Long., longitudinal*.

*P < 0.01 vs. Control.

*^†^*P* < 0.01 vs. Pre-operative MR*.

*^‡^*P* < 0.05 vs. Control*.

*^§^*P* < 0.05 vs. Pre-operative MR*.

*Age and gender effect are adjusted*.

### Mitral annular dynamics

**Figure 3:**
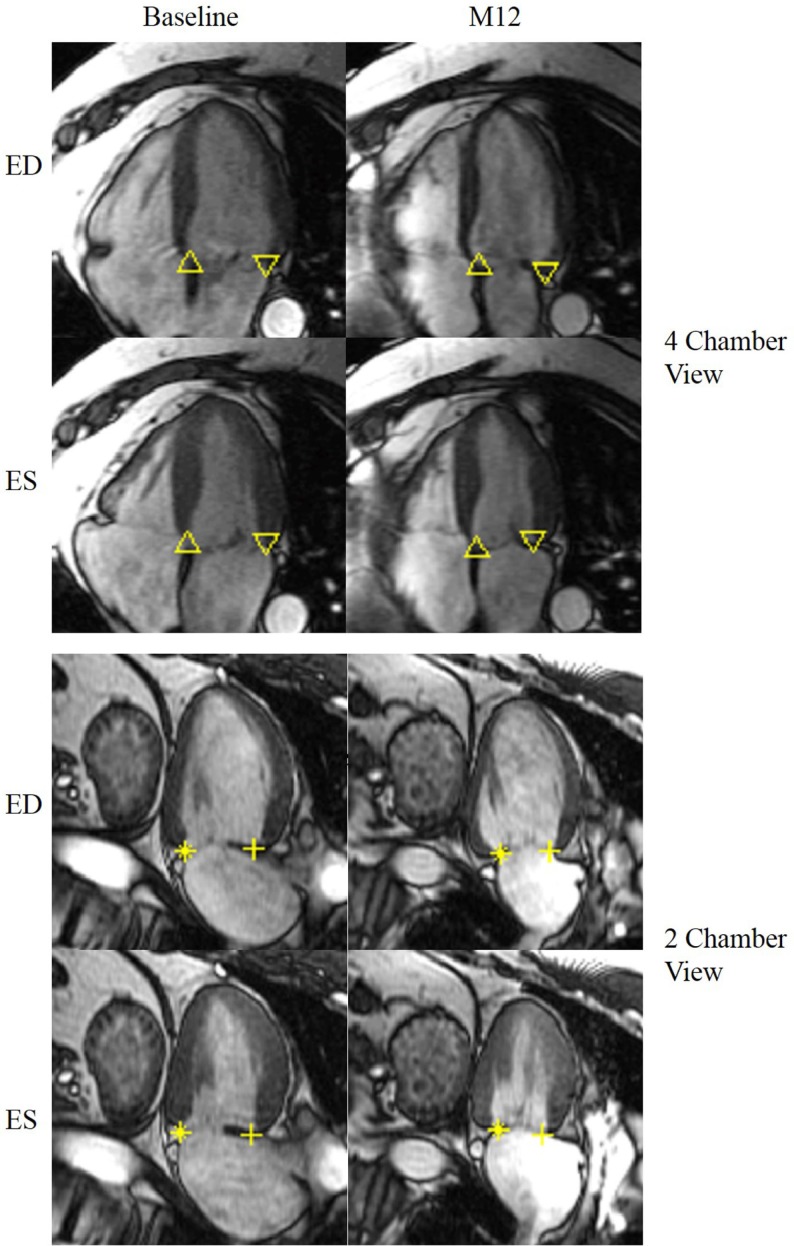
**Representative MR LV end-diastolic and end-systolic 4 chamber view (top) and 2 chamber view (bottom) before and after surgery**. The mitral annulus septal lateral diameter as well as the transverse diameter is reduced after surgery. Up triangle: basal septal; down triangle: basal lateral; + : basal anterior; *: basal inferior. ED: end-diastole; ES: end-systole.

**Figure 4:**
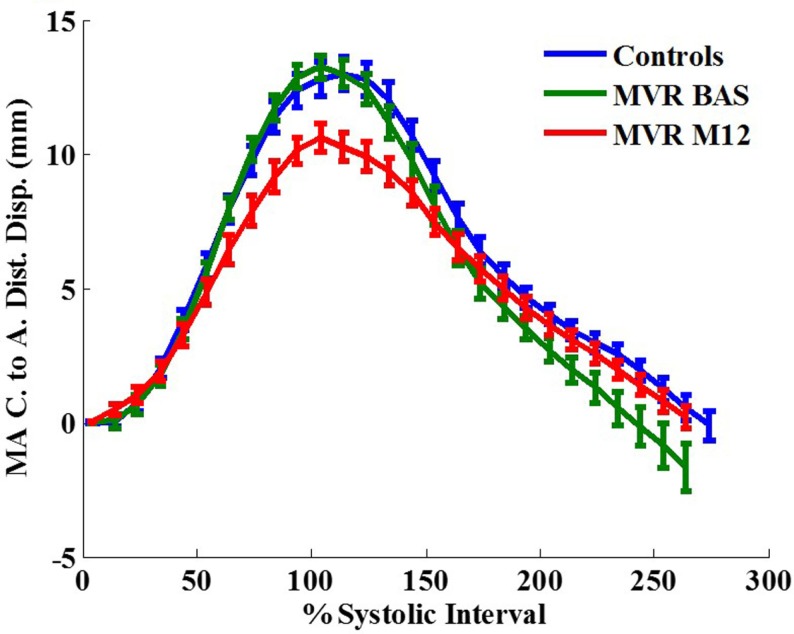
**Mitral annular centroid to apex shortening (in mm) vs. time curves in control vs. MR before and after surgery**. One year after surgery, MR patients demonstrate a decrease in mitral annulus mechanics compared with control. MA, mitral annulus; C, centroid; A, apex; Dist, distance; Disp, displacement. Data are mean ± SE.

Figure 3 demonstrates an example of MA landmarks of a randomly selected MR patient with four and two chamber views before and after surgery. Movies of this patient’s MA movements are attached as Supplemental Material. MA transverse diameter and septal-lateral diameter measured at ES were significantly dilated in MR at baseline vs. controls (transverse diameter: 45.83 ± 6.68 vs. 35.95 ± 4.07 mm, *P* < 0.001; septal-lateral diameter: 43.58 ± 7.52 vs. 35.51 ± 3.38 mm, *P* < 0.001), but returned to normal size after surgery due to the use of annuloplasty band (transverse diameter: 32.26 ± 5.1 mm, *P* < 0.001 vs. control and pre-surgical MR; septal-lateral diameter: 35.01 ± 5.27 mm, *P* < 0.001 vs. pre-surgical MR). However, MA dynamics in MR patients was impaired at 1-year post-surgery, demonstrated by the significant decrease in peak MA inferior, lateral, and septal displacements (Table 3). Furthermore, peak early diastolic MA lateral, septal, and centroid to apex relaxing rates were significantly decreased in post-surgical MR vs. control and pre-surgical MR. Figure 4 depicts the MA dynamics in terms of centroid to apex shortening-time curves in control and MR before and after surgery, indicating a decreased peak MA motion and flattened slope of diastolic arm of the curves in MR post-surgery, which correspond to the decreased peak early diastolic MA centroid to apex relaxation rate in MR after surgery.

**Table 3:** **MRI-derived mitral valve plane measurements**.

	Control (*n* *=* 51)	Mitral regurgitation	
		Preoperative (*n* *=* 35)	Postoperative (*n* *=* 35)
Peak MA anterior displacement (mm)	10.53 ± 3.09	11.71 ± 3.71	8.16 ± 2.8^†^
Peak E. Dia. MA anterior velocity (% LA length/s)	64.02 ± 25.1	76.74 ± 23.63*	55.19 ± 19.64^†^
Peak MA inferior displacement (mm)	12.73 ± 3.94	12.85 ± 3.52	8.6 ± 2.43*^,^^†^
Peak E. Dia. MA inferior velocity (% LA length/s)	64.93 ± 35.6	58.82 ± 21.97	39.83 ± 18.47^§^
Peak MA lateral displacement (mm)	13.38 ± 3.23	13.91 ± 3.09	10.39 ± 3.61*^,^^†^
Peak E. Dia. MA lateral velocity (% LA length/s)	79.32 ± 34.3	78.45 ± 24.73	49.4 ± 16.36*^,^^†^
Peak MA septal displacement (mm)	10.59 ± 3.55	12.04 ± 3.1	7.67 ± 2.44*^,^^†^
Peak E. Dia. MA septal velocity (% LA length/s)	71.26 ± 27.44	77.18 ± 39.92	43.03 ± 16.9*^,^^†^
Peak C. to A. Dist. displacement to ED (mm)	14.23 ± 4.31	14.47 ± 2.83	11.40 ± 3.10^‡,^^†^
Peak MA C. to A. Dist. shortening rate (mm/s)	72.73 ± 18.90	76.96 ± 20.40	58.87 ± 18.09^‡,^^†^
Peak E. Dia. MA C. to A. Dist. relaxation rate (mm/s)	69.62 ± 28.33	70.72 ± 22.82	44.18 ± 13.19*^,^^†^
Time to peak C. to A. shortening (ms)	340 ± 62	341 ± 58	331 ± 56

*Values are mean ± SD*.

*MA, mitral annulus; ES, end systole; C., mitral annulus plane centroid; A., apex; Dist., distance; E. Dia., early diastole; LA, long axis*.

*P < 0.01 vs. Control.

*^†^*P* < 0.01 vs. Pre-operative MR*.

*^‡^*P* < 0.05 vs. Control*.

*^§^*P* < 0.05 vs. Pre-operative MR*.

*Age and gender effect are adjusted*.

### LA volumetric and functional measurements

**Figure 5:**
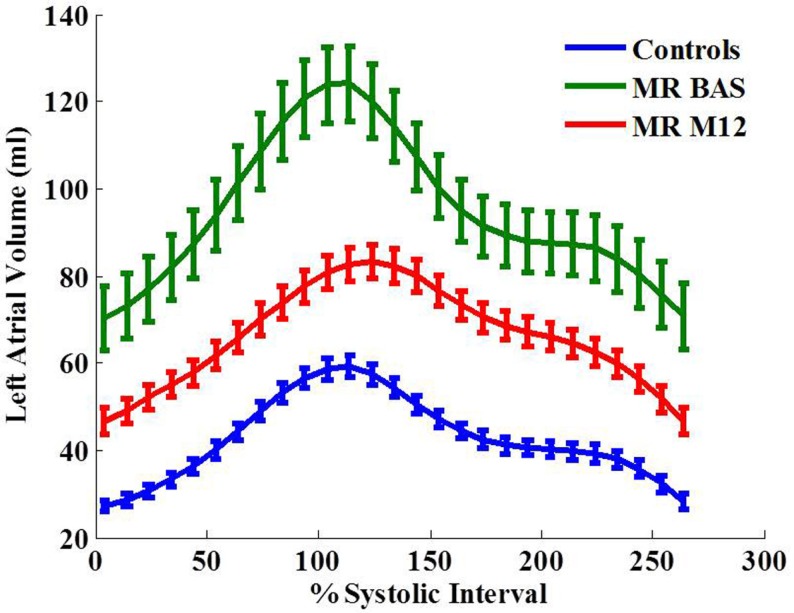
**Left atrial volume-time curves for the control group and MR group at baseline and 12 months after surgery**. Data are mean ± SE.

LA maximum, minimum volumes, and volume at atrial active kick were significantly increased in MR patients before surgery (Table 4; Figure 5). One year after surgery, the volumetric measurements were decreased but still significantly above normal except for LA maximum volume. LA early diastolic function measured as LAEF_passive_ and LA peak early diastolic ejection rate were significantly decreased below normal post-surgery. Time to maximum LA volume was increased post-surgery vs. its baseline.

**Table 4:** **MRI-derived LA volumetric and functional measurements**.

	Control (*n* *=* 51)	Mitral regurgitation	
		Pre-operative (*n* *=* 35)	Post-operative (*n* *=* 35)
LAV_min_ index (ml/m^2^)	14 ± 4	34 ± 19*	23 ± 8*^,^^†^
LAV_max_ index (ml/m^2^)	31 ± 8	61 ± 23*	40 ± 11^‡,^^†^
LAV_active_ index (ml/m^2^)	21 ± 6	44 ± 19*	32 ± 9*^,^^†^
LAEF	0.54 ± 0.08	0.45 ± 0.11^‡^	0.42 ± 0.10*
LAEF_active_	0.31 ± 0.10	0.23 ± 0.11*	0.28 ± 0.08
LAEF_passive_	0.32 ± 0.12	0.30 ± 0.09	0.20 ± 0.09*^,^^†^
LA Peak Filling Rate (LAV_max_/s)	2.54 ± 0.69	2.67 ± 0.70	1.99 ± 0.62
LA Peak E. Dia. Ejection Rate (LAV_max_/s)	3.11 ± 0.99	2.67 ± 0.88	1.80 ± 0.58*^,^^†^
Time to LAV_max_ (ms)	382 ± 53	354 ± 58^‡^	395 ± 46^†^

*Values are in mean ± SD*.

*LA, left atrium; LAV_max_ index, LA maximum volume at end-systole/body surface area; LAV_min_ index, LA minimum volume at end-diastole; LAV_active_ index, LA volume at atrial active kick; LAEF, LA ejection fraction; E. Dia., early diastole*.

*P < 0.01 vs. Control.

*^†^*P* < 0.01 vs. Pre-operative MR*.

*^‡^*P* < 0.05 vs. Control*.

*^§^*P* < 0.05 vs. Pre-operative MR*.

*Age and gender effect are adjusted*.

### Association of MA motion with LV, LA functions

Peak MA centroid to apex shortening was significantly associated with LVEF (*P* = 0.0059) and LAEF (*P* = 0.0156). During LV systolic phase, the linear relationships were positively significant between peak LV ejection rate and MA centroid to apex shortening rate (*P* = 0.0004) and between peak LA filling rate and MA centroid to apex shortening rate (*P* = 0.0093). During LV early diastolic phase, LV peak early diastolic filling rate was significantly associated with both MA peak early diastolic centroid to apex relaxation rate (*P* = 0.0113) and LA peak early diastolic ejection rate (*P* < 0.0001). All models were adjusted for age and gender.

## Discussion

The major findings of our current investigation are that one-year after surgery: (1) MA motion is impaired during diastole; (2) LV diastolic functions, comprehensively evaluated by cMRI, remain significantly impaired in MR; (3) LA volume and function do not return to normal post-surgery; and (4) MA motion is significantly associated with LV and LA systolic and diastolic functions. Our results therefore highlight the importance of further investigating the long-term effect of MA kinetics and its interaction with the LV and LA on the overall cardiac function post MV repair.

Mitral annulus plays an important role in LV function (19). In our current investigation, we find that both systolic displacement as well as early diastolic rebound of the MA is significantly reduced post-surgery. The performance of MA is significantly associated with both LV and LA systolic and diastolic functions. Previously it has been demonstrated that systolic MA excursion and shape variation track and contribute to global LV systolic function in the normal state (10). Since MA early diastolic rebound has significant contribution to early LV filling (20), impairment of this process is expected to reduce LV diastolic function. The precise mechanism of post-surgical MA dysfunction could not be delineated in this study. A number of factors may be affecting the MA motion, including the annuloplasty prosthesis itself, which superimposed upon inherent structural changes can impact the complex MA motion.

In our MR study cohort we note that using normalized diastolic variables, there are no significant LV functional differences in MR at baseline compared to normal controls. However, 1 year after MV repair, multiple diastolic variables are significantly decreased, indicating significantly impaired diastolic function. In addition to LV diastolic dysfunction after surgery, LA volumes do not return to normal and LAEF remains depressed as well as the LV early diastolic filling rate does. The abnormality of both LA and LV after surgery may imply a negative effect of MV repair in the coupling of LV and LA, in addition to intrinsic disease of the atrial myocardium. Another potential mechanism that may be participating in LV dysfunction post-surgery could be related to underlying myocyte dysfunction and myofibrillar degeneration as described previously (5). These myocellular changes may in part account for the reduction in early diastolic circumferential and longitudinal strain rates post-surgery.

The present study is limited in that the follow-up duration is only 1 year in a small number of well-characterized primary MR groups. The measurements of diastolic function utilizing cMRI are non-invasive. However, our cMRI measurements of systolic and diastolic function are very consistent among the two groups. All patients underwent MV repair using a variety of surgical techniques performed by an experienced surgeon. Due to small sample size, effect of different surgical techniques on mitral annular kinetics and cardiac function cannot be studied in the present study and is a topic for future research.

In conclusion, mitral annular motion, left atrial function, and LV diastolic strain rates are still impaired 1 year post MV repair. We have previously demonstrated impaired systolic function and persistent spherical LV remodeling 1 year after MV repair in the same patient cohort post MV repair (6). Long-term effects of these impairments on clinical and functional outcomes should be prospectively evaluated.

## Author Contributions

CS and HG were responsible for conception and design of the paper, and manuscript drafting and finalizing. CS, XZ, and TD were responsible for cine and tagged MRI image analysis, algorithm development and data generation. DM, MA, LD, SL, and HG were responsible for patient population design and recruiting. CS, LD, TD, SL, and IA were responsible for data generation and interpretation. CS, TD, DM, LD, and HG were responsible for revising paper critically for important intellectual content. Final approval of the manuscript was done by HG.

## Conflict of Interest Statement

The authors declare that the research was conducted in the absence of any commercial or financial relationships that could be construed as a potential conflict of interest.

## Supplementary Material

The Supplementary Material for this article can be found online at http://journal.frontiersin.org/article/10.3389/fcvm.2015.00031
